# 

**Published:** 1897-09

**Authors:** 


					﻿Southern Medical Record.
A Monthly Journal of Medicine and Surgery.
Vol. XXVII. ATLANTA, GA., SEPTEMBER, 1897.	No. 9^
BERNARD WOLFF, M. D., Editor.
Associate Editors :
A. W. GRIGGS, M. D.	WILLIS F. WFSTMORELAND, M. D.
j. mcfadden gaston, m. d.
E. Y. CLARKE, General Manager.	B. M. ZETTLER, Business Manager.
Entered at the Post-office at Atlanta, Ga., as Second-class Matter.
The Foote & Davies Co., Atlant*, Ga.
				

